# *Drosophila* as a Model to Study the Relationship Between Sleep, Plasticity, and Memory

**DOI:** 10.3389/fphys.2020.00533

**Published:** 2020-05-28

**Authors:** Stephane Dissel

**Affiliations:** Department of Molecular Biology and Biochemistry, School of Biological and Chemical Sciences, University of Missouri-Kansas City, Kansas City, MO, United States

**Keywords:** *Drosophila*, sleep, memory, plasticity, neurobiology

## Abstract

Humans spend nearly a third of their life sleeping, yet, despite decades of research the function of sleep still remains a mystery. Sleep has been linked with various biological systems and functions, including metabolism, immunity, the cardiovascular system, and cognitive functions. Importantly, sleep appears to be present throughout the animal kingdom suggesting that it must provide an evolutionary advantage. Among the many possible functions of sleep, the relationship between sleep, and cognition has received a lot of support. We have all experienced the negative cognitive effects associated with a night of sleep deprivation. These can include increased emotional reactivity, poor judgment, deficit in attention, impairment in learning and memory, and obviously increase in daytime sleepiness. Furthermore, many neurological diseases like Alzheimer’s disease often have a sleep disorder component. In some cases, the sleep disorder can exacerbate the progression of the neurological disease. Thus, it is clear that sleep plays an important role for many brain functions. In particular, sleep has been shown to play a positive role in the consolidation of long-term memory while sleep deprivation negatively impacts learning and memory. Importantly, sleep is a behavior that is adapted to an individual’s need and influenced by many external and internal stimuli. In addition to being an adaptive behavior, sleep can also modulate plasticity in the brain at the level of synaptic connections between neurons and neuronal plasticity influences sleep. Understanding how sleep is modulated by internal and external stimuli and how sleep can modulate memory and plasticity is a key question in neuroscience. In order to address this question, several animal models have been developed. Among them, the fruit fly *Drosophila melanogaster* with its unparalleled genetics has proved to be extremely valuable. In addition to sleep, *Drosophila* has been shown to be an excellent model to study many complex behaviors, including learning, and memory. This review describes our current knowledge of the relationship between sleep, plasticity, and memory using the fly model.

## Introduction

Sleep is an universal phenomenon that has been described in a variety of species ranging from worms to humans (Keene and Duboue, 2018). In addition to animals with complex and organized nervous systems, recent studies have also described sleep in models with simpler nervous systems, such as jellyfish (Nath et al., 2017). At first look, sleep could appear to be a detrimental behavior. This is because when animals are sleeping they are not gathering food or attending to their progeny, they also are not performing vital evolutionary functions like reproduction and perhaps more importantly they are subject to predation. However, despite all the negative outcomes attached to it, sleep has been maintained throughout evolution, emphasizing its essential value (Miyazaki et al., 2017). This nearly ubiquitous presence of sleep in the animal kingdom strongly supports the view that sleep must provide an evolutionary advantage and perform a vital function for the organism. While this has been widely accepted by the scientific community, it is worth mentioning that a recent study challenged the notion that sleep performs a vital function (Geissmann et al., 2019).

Over the last 100 years, the fruit fly, *Drosophila melanogaster* has been used as a model to study many biological questions. The unparalleled genetic tools, cost effectiveness, short developmental time and relevance to human physiology of the fly has positioned it as a prominent model organism. One particular aspect of biology where *Drosophila* contributed extensively is neurobiology. Starting with the discovery of the *period* gene in 1971 by Konopka and Benzer (1971), *Drosophila* has been a workhorse in the study of complex behaviors, such as courtship (Yamamoto and Koganezawa, 2013), aggression (Kravitz and Fernandez, 2015), feeding (Bhumika and Singh, 2018), drug addiction (Kaun et al., 2012), learning and memory (Kahsai and Zars, 2011), circadian rhythms (Franco et al., 2018), and sleep (Ly et al., 2018).

While *Drosophila* has been the pioneer model used to elucidate the molecular mechanisms underlying circadian rhythms, leading to the 2017 Nobel Prize in Physiology and Medicine awarded to Hall, Rosbash and Young, whether flies displayed a behavioral state similar to mammalian sleep was unclear for many years. It is only in 2000, that two independent groups clearly demonstrated that fruit flies were indeed sleeping (Hendricks et al., 2000; Shaw et al., 2000).

Sleep is highly sensitive to internal and external factors and can be modulated accordingly to satisfy an individual’s need. For example, in great frigatebirds, sleep manifests itself in a very different manner whether the birds are flying or on land (Rattenborg et al., 2016). Furthermore, some animals can function without sleep for various time durations, such as in neonates killer-whales and their mother during the first postpartum month (Lyamin et al., 2005) or in elephants if falling asleep puts them at risk of being killed (Gravett et al., 2017). In humans, sleep is not as efficient when sleeping in unfamiliar surroundings (Agnew et al., 1966). This so-called first-night effect appears to be caused by the fact that one hemisphere of our brain is more vigilant than the other when we sleep in a novel environment, probably reflecting a protective mechanism (Tamaki et al., 2016).

Beyond environmental and external stimuli, internal stimuli can also modulate sleep. For example, sleep deprivation leads to a homeostatic sleep rebound, illustrated by an increase in sleep quantity and depth following deprivation (Borbely, 1982). This increase in sleep is especially due to an increase in slow-wave sleep (Dijk et al., 1987). Finally, sleep interacts with many biological functions. For instance, there are bidirectional links between sleep and immunity, with immune system activation capable of modulating sleep (Besedovsky et al., 2019) and between diet/metabolism and sleep (Huang et al., 2011; Frank et al., 2017).

In this review, I will describe sleep in the *Drosophila* model and introduce the many brain regions involved in sleep regulation. I will then address the relationships between sleep and plasticity and between sleep and memory.

## Sleep in *Drosophila*

Daily rhythms of rest/activity in flies have been extensively studied starting in the 1970’s (Konopka and Benzer, 1971). Under laboratory conditions, flies are crepuscular animals displaying two peaks of activity centered on the dark to light and light to dark transitions. However, it remained unclear until the year 2000 whether the rest observed in flies could be considered as sleep or whether it was simply inactivity. From a behavioral point of view, human sleep is a period of reduced activity; it is associated with a typical posture, such as lying down; it leads to a reduction in responsiveness to mild external stimuli but it can be easily reversed if the stimuli is strong enough; that is sleep is different from other states of reduced responsiveness like coma. Mammalian sleep is also characterized by a change in brain electrical activity that can be measured by electroencephalography (EEG). Importantly, sleep is regulated by two processes, a circadian process that dictates when sleep can occur and a homeostatic process that controls how much sleep an individual needs (Borbely, 1982).

In *Drosophila*, assessing sleep using an electrophysiological criteria is a challenging task. Thus, in order to unequivocally characterize sleep in the fly model, two independent groups relied on a behavioral definition of sleep (Hendricks et al., 2000; Shaw et al., 2000). These groundbreaking studies demonstrated that locomotor inactivity lasting at least 5 min was associated with an increased arousal threshold, as assessed using mild mechanical stimulation. However, if the stimulus was strong enough, flies that had been inactive for 5 min or more would respond (Hendricks et al., 2000; Shaw et al., 2000). Importantly, both studies also found that the rest observed in flies was homeostatically regulated. Following rest deprivation, flies showed an increase in rest (Hendricks et al., 2000; Shaw et al., 2000). Video analysis of *Drosophila* rest/activity behavior revealed that flies adopt a specific posture to engage in rest periods. Moreover, they do so in a specific location within the tubes where they are housed (Hendricks et al., 2000). Additionally, rest is abundant in young flies and reduced in older flies; an observation that parallels what we see in human sleep (Shaw et al., 2000). Rest is also modulated by stimulants and hypnotics (Hendricks et al., 2000; Shaw et al., 2000). The observations made in these two studies demonstrated that periods of rest lasting 5 min or more satisfy all the behavioral hallmark of sleep in humans, a typical posture, and withdrawal from the environment, higher arousal threshold and homeostatic regulation (Hendricks et al., 2000; Shaw et al., 2000). In addition, in *Drosophila* like in mammals, sleep is sexually dimorphic; male flies sleep more than female, especially during the day (Isaac et al., 2010; Khericha et al., 2016; Wu et al., 2018). Interestingly, sleep is also present in larvae, and is important for neurogenesis (Szuperak et al., 2018). Because of the strength of the *Drosophila* model, these seminal studies gave rise to new avenues of research that could help understand our knowledge of sleep regulation and function.

Although the definition of sleep in flies is a behavioral one, local field potential recordings demonstrated that sleep is a state of reduced neuronal activity (Nitz et al., 2002). Later studies, using the calcium indicator GCaMP, confirmed this electrophysiological observation and also reinforced the notion that sleep is a state of reduced behavioral responsiveness (Bushey et al., 2015).

Not surprisingly, in the years following the characterization of sleep in *Drosophila*, many progresses have been made regarding the genes that regulate sleep. Importantly, these studies demonstrated that the signaling mechanisms that regulate sleep are conserved between the fly and mammalian models (Sehgal and Mignot, 2011). For example, the role of monoamines in sleep regulation is similar in *Drosophila* and mammals (for instance, dopamine promotes wakefulness while serotonin promotes sleep in both models; Nall and Sehgal, 2014).

## Brain Regions Modulating Sleep in *Drosophila*

**FIGURE 1:**
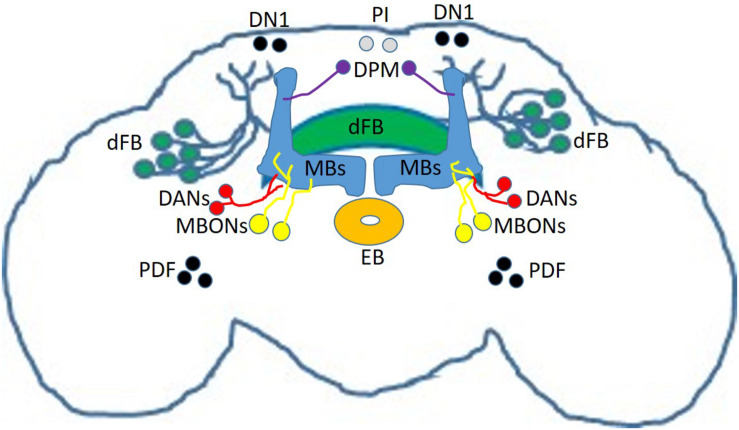
Sleep-regulating circuits in the fly brain. Schematic representation of neurons and circuits involved in sleep regulation including Mushroom Bodies (MBs, blue); Mushroom Body Output Neurons (MBONs, yellow); dopaminergic neurons (DANs, red); circadian clock neurons (black) including Pigment-Dispersing Factor (PDF) neurons and Dorsal Neurons 1 (DN1); Ellipsoid Body neurons (EB, orange); dorsal Fan-Shaped Body neurons (dFB, green); Pars Intercerebralis neurons (PI, gray) and Dorsal-Paired Medial Neurons (DPM, purple).

There are many brain regions involved in sleep regulation in the mammalian brain (Saper and Fuller, 2017). Similarly, the fly brain contains many sleep regulating centers. Chronologically, the Mushroom Bodies (MBs) was the first identified by two independent groups in 2006 (Joiner et al., 2006; Pitman et al., 2006). MBs are essential bilateral structures in the fly brain involved in learning and memory (Figure 1, blue; Hige, 2018). The Kenyon Cells (KCs), neurons intrinsic to the MBs synapse on to 21 different types of Mushroom Body Output Neurons (MBONs, Figure 1, yellow) to modulate attraction or avoidance (Aso et al., 2014a, b). These KCs-MBONs connections are modulated by dopaminergic neurons (DANs, Figure 1, red). In addition to learning and memory, the MBs and its associated MBONs and DANs regulate sleep; some KCs-MBONs connections promote sleep while other promote wake (Aso et al., 2014b; Sitaraman et al., 2015a, b).

Sleep is regulated by both a circadian process and a homeostatic process (Borbely, 1982), so perhaps it is not surprising that neurons regulating circadian rhythms are also capable of modulating sleep. Among the roughly 150 clock cells in the fly brain (Franco et al., 2018), Pigment-Dispersing factor (PDF) lateral neurons (Renn et al., 1999) play an essential role in controlling rhythmic locomotor activity (Grima et al., 2004; Stoleru et al., 2004). In addition to their central role in controlling the clock, studies demonstrated that PDF cells (Figure 1, black) are promoting wake (Parisky et al., 2008; Shang et al., 2008; Sheeba et al., 2008). Interestingly, PDF neurons are important for memory induced by courtship conditioning (Donlea et al., 2009). Furthermore, more recent studies demonstrated that another group of clock neurons, the Dorsal Neurons 1 (DN1s, Figure 1, black) can modulate sleep (Kunst et al., 2014; Guo et al., 2016, 2017; Lamaze et al., 2017), by making synaptic connections with tubercular-bulbar (TuBu) neurons (Guo et al., 2018; Lamaze et al., 2018). These TuBu neurons, are forming synaptic connections with R-neurons innervating the ellipsoid body (EB, Figure 1, orange) (Guo et al., 2018; Lamaze et al., 2018), a region involved in the control of homeostatic sleep drive (Liu et al., 2016). These data provide an anatomical and functional link between clock controlling neurons and sleep regulating centers.

The Fan-Shaped Body (FB) is part of the central complex (CX) in the *Drosophila* brain. It is a region organized into multiple layers that plays a role in many functions, including locomotion control (Strauss, 2002), courtship behavior (Sakai and Kitamoto, 2006) and memory (Joiner and Griffith, 1999; Sakai et al., 2012), nociception (Hu et al., 2018), visual feature recognition (Liu et al., 2006) and processing (Weir and Dickinson, 2015), and social behaviors (Kacsoh et al., 2019). Neurons that project to the dorsal Fan-Shaped Body (dFB, Figure 1, green) are sleep promoting (Donlea et al., 2011), and acute activation of these neurons can help convert short-term memory (STM) into long-term memory (LTM) (Donlea et al., 2011). However, a recent study questions whether this memory benefit is actually caused by dFB or by ventral Fan-Shaped Body (vFB) neurons activation (Dag et al., 2019). Nevertheless, vFB neurons can also promote sleep when activated, confirming that the FB is an important sleep-regulating hub in the fly brain (Dag et al., 2019). The dFB is particularly important for the homeostatic regulation of sleep (Donlea et al., 2014). The electrical activity of dFB is modulated by sleep pressure, a point illustrated by the fact that sleep deprivation increases the excitability of dFB neurons (Donlea et al., 2014). dFB neurons are modulated by dopaminergic inputs (Liu et al., 2012; Ueno et al., 2012; Pimentel et al., 2016; Ni et al., 2019) with dopamine capable of switching dFB neurons from an electrically active ON state to an electrically silent OFF state (Pimentel et al., 2016). Importantly, this switching mechanism is reminiscent of the FLIP-FLOP switching model between sleep and wake in mammalian brains (Saper et al., 2010). Further work demonstrated that dFB neurons form inhibitory synaptic connections with helicon cells (Donlea et al., 2018). Interestingly, helicon cells themselves provide excitation to R2 neurons of the EB (Donlea et al., 2018), which as mentioned previously control homeostatic sleep drive (Liu et al., 2016).

Other regions regulating sleep include the Pars intercerebralis (PI, Figure 1, gray), a neuroendocrine structure in the fly brain (Foltenyi et al., 2007; Crocker et al., 2010). Interestingly, the PI is connected to the clock network and is an important component of the circadian output pathway for rest/activity rhythms (Cavanaugh et al., 2014). In addition, the dorsal paired medial (DPM, Figure 1, purple) neurons, which innervate the MBs and are involved in memory consolidation (Waddell et al., 2000; Keene et al., 2004), are sleep promoting cells (Haynes et al., 2015). Finally, and confirming data obtained from mammalian models (Halassa et al., 2009), glia is involved in sleep regulation in *Drosophila* (Seugnet et al., 2011b; Chen et al., 2015; Dissel et al., 2015c; Farca Luna et al., 2017; Gerstner et al., 2017b; Vanderheyden et al., 2018).

These data illustrate that similarly to the mammalian brain, the fly brain contains many regions that regulate sleep. Interestingly, many of these are also involved in learning and memory positioning them perfectly to modulate the interaction between these two processes.

## Sleep and Plasticity

As mentioned previously, sleep is a plastic behavior, it is modulated by both internal, and external/environmental stimuli. Examples include the bidirectional relationship between sleep and the immune system (Williams et al., 2007; Dissel et al., 2015c; Toda et al., 2019). Interestingly, both neurons and glia are implicated in this process (Dissel et al., 2015c) with different outcomes in learning and memory. Another example is the strong bidirectional link between metabolism/diet and sleep (Catterson et al., 2010; Keene et al., 2010; Thimgan et al., 2010; Murphy et al., 2016; Yurgel et al., 2019). Because the focus of this review is on sleep and plasticity/memory, these relationships won’t be described further but are nevertheless very important.

During wakefulness, when animals are performing their daily behavioral tasks, for instance exploring their surroundings, reacting to sensory stimuli, interacting with other individuals, making decisions, forming memories, they learn about their environment. These waking experiences are the behavioral basis of learning and memory. Within the brain, learning and memory can be seen as long-lasting changes in the strength or number of synaptic connections between neurons (Tononi and Cirelli, 2014). Importantly, sleep is influenced by neural activity and plasticity (Tononi and Cirelli, 2014). One of the prominent theories to explain the function of sleep is the synaptic homeostasis hypothesis (SHY) (Tononi and Cirelli, 2003). In the SHY model, the function of sleep is the downscaling of synaptic connections that have been strengthened during previous waking experiences (Tononi and Cirelli, 2003). Importantly, the SHY model has received experimental support in the mammalian brain (Huber et al., 2013; de Vivo et al., 2017; Diering et al., 2017; Li et al., 2017; Norimoto et al., 2018). However, it is important to note that synaptic potentiation has also been observed during sleep (Frank et al., 2001; Aton et al., 2013, 2014; Seibt and Frank, 2019). Nevertheless these data reinforce the strong relationship between synaptic plasticity and sleep.

Waking experiences modulate sleep and sleep and neuronal plasticity have a bidirectional relationship (Tononi and Cirelli, 2014). This is also true in flies. Obviously, the most important internal factor than can modulate sleep is sleep pressure. Following sleep deprivation, animals are subject to a strong drive to increase the duration and depth of sleep. The same observation was made in flies, where sleep deprivation triggers a homeostatic sleep rebound (Hendricks et al., 2000; Shaw et al., 2000; Huber et al., 2004). Studies have demonstrated that the dFB and the EB are important circuits regulating sleep homeostasis in *Drosophila* (Donlea et al., 2014, 2018; Liu et al., 2016). Importantly, dFB and EB interact to regulate sleep homeostasis (Liu et al., 2016; Donlea et al., 2018).

Extended waking periods not only increase sleep pressure, they also increase the strength of synaptic connections (Tononi and Cirelli, 2014). This potentiation of synaptic connections constitutes the mechanism that underlies learning and memory. In *Drosophila*, waking, whether spontaneous or imposed by sleep deprivation, led to an increase in several key synaptic proteins in the brain (Gilestro et al., 2009; Dissel et al., 2015a). Importantly, the level of these synaptic markers was reduced following sleep (Gilestro et al., 2009; Dissel et al., 2015a). Reinforcing this notion is the finding that the number and size of synapses in three different circuits, including MBs and PDF neurons, increases following wake and decreases following sleep (Donlea et al., 2009; Bushey et al., 2011). While these data support the hypothesis that sleep plays a role in downscaling synaptic connections that have been strengthened during previous waking experiences (the SHY model) (Tononi and Cirelli, 2003) in *Drosophila*, it is important to note that sleep has been found to potentiate some synaptic connections. This is especially important in the developing brain (Frank et al., 2001). Newborn babies spend a lot of time sleeping and this sleep is very important for their development (Bathory and Tomopoulos, 2017). Similarly, and as previously mentioned, newly hatched flies sleep a lot in the first days of their life (Shaw et al., 2000). This early-life sleep plays an essential role in brain development. Studies have demonstrated that early-life sleep deprivation caused long lasting behavioral defects that are linked to impaired development in key brain areas (Seugnet et al., 2011a; Kayser et al., 2014). Taken together, these data clearly demonstrate that sleep and neuronal plasticity are tightly interconnected. However, they also suggest that this relationship is not unidirectional, sleep could both downscale and potentiate specific types of synaptic connections. Supporting this view are data collected in a classical memory mutant, where induction of sleep could restore learning and memory as well as increase levels of a key synaptic protein (Dissel et al., 2015a). It is thus very likely that sleep can modulate plasticity in both directions, in a manner adapted to specific needs or circuits.

While awake, *Drosophila* engage in a variety of behaviors, ranging from simple motor actions to very complex social interactions. One such waking experience is social enrichment, which consists of housing many flies within a single vial, increasing the likelihood of social interactions. Flies that are kept in a socially enriched environment display an increase in synapse numbers in the PDF expressing large Lateral Neurons (Donlea et al., 2009) and sleep more than flies that are socially isolated (Ganguly-Fitzgerald et al., 2006). Importantly, PDF cells are essential for the sleep increase triggered by social enrichment (Donlea et al., 2009).

Other waking experiences include different types of memory training. In *Drosophila*, such a memory training is courtship conditioning. In courtship conditioning, naïve males learn to suppress their drive to court by pairing them with non-receptive females (Griffith and Ejima, 2009). This training protocol can create long lasting memories that are dependent on the MBs (McBride et al., 1999). Importantly, courtship conditioning protocols that create LTM increase post-training sleep (Ganguly-Fitzgerald et al., 2006; Dag et al., 2019), and this post-training sleep is essential for the manifestation of LTM (Ganguly-Fitzgerald et al., 2006; Dag et al., 2019). Interestingly, during post-training sleep, the neurons that were engaged in memory acquisition are reactivated and this reactivation is essential for LTM formation (Dag et al., 2019). This finding parallels the memory consolidation processes observed in mammals (Stickgold, 2005; Born, 2010).

Altogether, these data clearly support the notion that sleep is plastic, and that sleep and neuronal plasticity mutually influence each other. Uncovering the molecular mechanisms underlying this relationship is an essential aspect of neurobiology that will help us understand how the brain can optimize its functions in oscillating behavioral states.

## Sleep and Memory

We spend nearly a third of our life asleep, but despite years of research in humans and animal models, we still do not know why we sleep. The function, or probably the functions of sleep remain one of neuroscience biggest mystery (Krueger et al., 2016). Such an enigma has obviously attracted the curiosity of countless number of scientists and many theories to explain the function of sleep have been proposed over the years (Krueger et al., 2016). While some have more merits than others, one of the most attractive one is the one pertaining to the influence of sleep on learning and memory (Chen and Wilson, 2017). That is, on one hand, sleep plays a positive role in memory consolidation (Pavlides and Winson, 1989; Wilson and McNaughton, 1994; Walker and Stickgold, 2004; Stickgold, 2005; Born, 2010) while sleep deprivation and sleep disruption impairs learning and memory (Killgore, 2010; Havekes et al., 2015; Krause et al., 2017). In the brain, learning and memory can be observed at the level of synaptic connections between neurons. Importantly, sleep has been demonstrated to strongly modulate synaptic plasticity (Diekelmann and Born, 2010).

The relationship between sleep and memory can be investigated in flies that have spontaneously reduced levels of sleep or in wild-type flies that are sleep deprived (Dissel et al., 2015b). Flies with spontaneously fragmented sleep are STM impaired as assessed with Aversive Phototaxic Suppression assay (APS) (Le Bourg and Buecher, 2002; Seugnet et al., 2008, 2009a). In the APS, an individual fly is inserted in a T-maze and has a choice between two paths leading to two independent vials. One of them is illuminated while the other is in dark. The lighted portion of the maze contains an aversive stimulus, quinine. Once the fly has made a choice (light or dark), it is removed from the maze and reinserted at the entry point. Each individual fly goes through the maze 16 consecutive trials. At first, wild-type flies will go to the lighted vial where they will encounter the aversive stimulus. As the training progresses, flies will start to visit the dark vial more often. Performance in the APS is calculated as the percentage of dark choices in the last 4 trials of a training session. Typically, wild-type flies never make more than one visit to the dark vial in the last 4 trials if no quinine is present on the lighted vial. Thus, a performance index close to or superior to 50% (at least 2 dark visits in the last 4 trials) indicate learning (Seugnet et al., 2009a). Interestingly, performance in the APS requires the expression of the dopamine D1 receptor in the MBs (Seugnet et al., 2008).

Some studies have looked at memory impairments in mutant flies that display reduced sleep. Loss of function mutations in the ß modulatory subunit of the Shaker potassium channel, encoded by the *Hyperkinetic* (*Hk*) gene reduce sleep (Bushey et al., 2007). Interestingly, when tested for STM using the heat-box paradigm, *Hk* mutants were impaired (Bushey et al., 2007). The heat-box is an operant conditioning paradigm where flies learn and remember to avoid one-half of a dark chamber associated with a temperature that is aversive (Wustmann et al., 1996; Diegelmann et al., 2006). Another study demonstrated that mutations in the Rho-GTPase-activating protein encoded by the *crossveinless-c* (*cv-c*) gene lead to decreased sleep time and STM deficits as assessed with aversive olfactory conditioning (Donlea et al., 2014). In aversive olfactory classical conditioning, the fly learns to associate an odor and a mild electric shock (Krashes and Waddell, 2011).

In an effort to develop a fly model of insomnia, an insomnia-like strain was created by crossing short sleeping flies together for 60 generations (Seugnet et al., 2009b). These flies only slept 60 min a day and were memory impaired as assessed with the APS (Seugnet et al., 2009b).

These data indicate that a reduction in sleep quantity and quality can lead to learning and memory impairments, however, it is important to note that this is not always the case. For example, when looking at individual flies within a wild-type stock, one can find individuals with fragmented sleep that have normal performance using the APS (Dissel et al., 2015c). Thus, these individuals have developed resilience to the negative effects of sleep degradation (Dissel et al., 2015c).

The remarkable capacity of sleep to benefit learning and memory was further demonstrated when sleep was increased in the classical memory mutants *rutabaga* (*rut*) and *dunce* (*dnc*) (Dissel et al., 2015a). Sleep was increased using three independent strategies (feeding the flies the GABA-A agonist THIP, genetic activation of the dFB and increasing the expression of Fatty acid binding protein) prior to APS training in both mutants. Surprisingly, both memory mutants could form STM following sleep induction (Dissel et al., 2015a). Furthermore, the capacity to form LTM as assessed with courtship conditioning was also restored by sleep induction before and after memory training (Dissel et al., 2015a). Importantly, THIP has been validated as a potent sleep-promoting drug in several subsequent studies (Berry et al., 2015; Dissel et al., 2017; Stahl et al., 2017; Yap et al., 2017; Artiushin et al., 2018; Hill et al., 2018; Ki and Lim, 2019; Liu et al., 2019). In particular, inducing sleep with THIP was shown to block dopamine neuron mediated forgetting of olfactory memories (Berry et al., 2015).

Neurodegenerative diseases like Alzheimer’s disease (AD) are often accompanied with sleep deficits (Wang and Holtzman, 2019). In order to study this destructive disease, several fly models of AD have been developed over the years (Finelli et al., 2004; Greeve et al., 2004; Iijima et al., 2004; McBride et al., 2010; Chakraborty et al., 2011; Mhatre et al., 2013; Mhatre et al., 2014), and it was demonstrated that sleep is disrupted in some of these models (Dissel et al., 2015a; Tabuchi et al., 2015; Farca Luna et al., 2017; Gerstner et al., 2017a; Song et al., 2017; Buhl et al., 2019). Importantly, inducing sleep with THIP can restore memory in several fly models of Alzheimer’s disease (Dissel et al., 2015a, 2017). Thus, inducing sleep can help the brain overcome memory deficits created by critical genetic lesions or neurodegenerative processes.

In addition to looking at flies with naturally occurring low levels of sleep, the relationship between sleep and learning/memory in *Drosophila* can be studied by looking at the effect of sleep deprivation (SD) on subsequent performance. Following SD, performance, measured as an escape response to an aversive stimulus made of a combination of noise and vibration was reduced (Huber et al., 2004). Furthermore, using the APS or aversive olfactory classical conditioning, it was demonstrated that following SD, wild-type flies are impaired (Seugnet et al., 2008; Li et al., 2009).

In addition to SD, it is possible to reduce sleep by increasing the activity of wake-promoting neurons. Activation of wake-promoting neurons results in subsequent STM impairments as assessed with an aversive-taste memory paradigm (Seidner et al., 2015). In aversive-taste memory, flies learn to suppress their proboscis extension reflex in response to the simultaneous pairing of appetitive fructose to the tarsi and aversive quinine at their proboscis (Keene and Masek, 2012). Importantly, the formation of aversive taste memory is dependent on the MBs.

Sleep is also important for the consolidation of recently acquired memories in long lasting LTM (Walker and Stickgold, 2004; Stickgold, 2005). For instance, a few hours of SD immediately following a courtship conditioning protocol that induces LTM blocked the formation of LTM (Ganguly-Fitzgerald et al., 2006; Dag et al., 2019). Furthermore, increasing sleep following a courtship protocol that normally only creates STM can create LTM (Donlea et al., 2011; Dissel et al., 2015a; Dag et al., 2019). These data suggest that sleep can help the brain recruit mechanisms that are beneficial for the formation of LTM, further illustrating the positive role that sleep plays in memory processing.

## Conclusion

The nearly ubiquitous presence of sleep in the animal kingdom, despite the obvious detrimental consequences of being in this behavioral state, suggests that sleep must serve an absolutely vital function for the organism. However, despite many efforts, the function of sleep remains elusive. Among the many possible function of sleep, the bidirectional relationship between sleep and plasticity/memory has been extensively documented. Sleep is important for optimal cognitive performance, and sleep disruptions lead to defects in learning and memory. Conversely, many experiences that change plasticity and memory modulate sleep. In addition, neurodegenerative diseases like Alzheimer’s disease often disrupts sleep. Thus, it is clear that understanding the sleep-plasticity-memory relationship could have major therapeutic impacts.

In that respect, *Drosophila* seems to be the perfect model. With its considerable strength as a genetic model, coupled with low cost and fast generation time, and almost constant technological advances, progress can be made in *Drosophila* in unparalleled ways. Importantly, the relationship between sleep and memory has been mostly characterized using a “what goes wrong in the brain when sleep is disrupted?” strategy using sleep deprivation, or mutations that disrupt sleep. This approach has proved to be extremely valuable but maybe it only gave us partial answers. With the ability to induce sleep on demand, one can now ask the following question “what good does sleep do to the brain?” While these two questions may look similar, the answers to them may be complementary and could help us get a better understanding of the function of sleep. In conclusion, the strength of the *Drosophila* model will be invaluable to help us understand how sleep can benefit cognitive processes in the context of health and diseases. This could prove especially important in the case of neurodegenerative diseases like Alzheimer’s disease.

## Author Contributions

The author confirms being the sole contributor of this work and has approved it for publication.

## Conflict of Interest

The author declares that the research was conducted in the absence of any commercial or financial relationships that could be construed as a potential conflict of interest.
